# Similarities and differences in carotid artery, hemodynamic, and autonomic reactivity induced by mental stress or cold in adults: A randomized crossover study

**DOI:** 10.14814/phy2.70803

**Published:** 2026-03-11

**Authors:** Francisco J. Fensterseifer, Iara G. Teixeira, Welliton Oracz, Richard E. Filipini, Francine Stein, Guilherme F. Speretta

**Affiliations:** ^1^ Multicenter Post‐Graduate Program in Physiological Sciences, Biological Sciences Center Federal University of Santa Catarina Florianópolis SC Brazil; ^2^ Post‐Graduate Program in Neurosciences, Biological Sciences Center Federal University of Santa Catarina Florianópolis SC Brazil; ^3^ Department of Physiological Sciences, Biological Sciences Center Federal University of Santa Catarina Florianopólis Brazil

**Keywords:** cardiovascular risk, cold pressor test, heart rate variability, physical stress, Stroop color and word test

## Abstract

The cold pressor test (CPT) is used to investigate cardiovascular and autonomic responses, while the Stroop color‐word test (Stroop) is also used for this purpose, although its vascular effects require further understanding. This randomized crossover study compared vascular, hemodynamic, and autonomic responses induced by cold and mental stress. Twenty participants (10 females; 25.7 ± 5.2 years) underwent the Stroop and CPT. Carotid artery reactivity (CAR) was evaluated using ultrasound. Arterial pressure (AP), heart rate (HR), and R‐R intervals (RRi) were continuously recorded. RRi were used to infer autonomic modulation through HR variability. Parametric data are reported as mean ± SD and non‐parametric data as median (p25‐p75). CAR magnitude did not differ between tests (Stroop: 2.8 ± 3.6; CPT: 4.1 ± 4.2%; p = 0.236), although Stroop elicited a shorter time to peak (66.5 ± 56.5 vs. 99.5 ± 35.1 s; *p* = 0.020). CPT induced a greater rise in diastolic AP (Stroop: 8.5 (6.2–10.0); CPT: 13.0 (6.2–23.5) mmHg; *p* = 0.009) and mean AP (Stroop: 8.4 ± 4.2; CPT: 13.5 ± 8.5 mmHg; *p* = 0.023). Stroop produced a larger HR increase (20.5 (10.2–27.7) vs. 11.0 (5.2–14.7) bpm; *p* = 0.013) and larger decrease in parasympathetic modulation (RMSSD – Stroop: −21.3 (−39.6 to −10.0); CPT: −12.8 (−22.8 to 0.95) ms; *p* = 0.026; HF – Stroop: −2.3 ± 1.3; CPT: −0.61 ± 0.74 Ln ms^2^; *p* < 0.001). In summary, Stroop evoked a CAR similar in magnitude to CPT, but with an earlier peak, supporting anticipatory physiological adjustments to mental stress.

## Graphical abstract

The Stroop test and cold pressor test elicit comparable common carotid artery vasodilation. However, mental stress induced by the Stroop test leads to an earlier peak response, possibly influenced by anticipatory mechanisms. Created in BioRender. Speretta, G. (2026) https://BioRender.com/mwpc1hw.

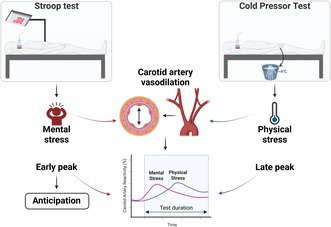

## 1 INTRODUCTION

Stress can be defined as a state of tension caused by a challenging situation (World Health Organization, 2024). Stressful events trigger a complex set of adaptive physiological and behavioral responses that seek to reestablish the challenged body's homeostasis. The sympathetic nervous system (SNS) and the hypothalamic–pituitary–adrenal (HPA) axis constitute the main effector pathways in the stress response (Godoy et al., 2018; Ulrich‐Lai & Herman, 2009). The SNS stimulates the medulla of the adrenal glands to release epinephrine and norepinephrine. These hormones, together with neurotransmitters released by sympathetic postganglionic neurons, increase heart rate (HR) and the force of myocardial contraction, besides promoting vasoconstriction or vasodilation in arteries and arterioles, acting on α or β receptors, respectively, to redistribute blood flow to essential tissues such as muscles and brain. All these responses increase arterial pressure (AP) (Kivimäki & Steptoe, 2018; Barbato, 2009). The activity of the other arm of the autonomic nervous system (ANS), the parasympathetic nervous system (PNS), is usually reduced during stress, although in some cases it may also be activated to counteract the effects of SNS on blood pressure (Carrive, 2006; Ulrich‐Lai & Herman, 2009).

Stressful situations may arise from physical, psychological, or combined stimuli. Factors such as pain, injury, or intense physical exertion are generally considered physical stress, while mental stress is related to emotional or cognitive challenges, such as challenging social situations (Lundberg, 2006). Particularly stressful mental situations may lead to an anticipatory response, often interpreted as allostasis, which is the capacity of a biological system to allow dynamic adaptations to environmental challenges, adjusting physiological parameters as necessary (Goldstein & Allostasis, 2002; McEwen & Wingfield, 2003).

The cold pressor test (CPT) is a widely used stressor, first reported in 1936 (Hines & Brown, 1936), which consists of immersing one extremity of the body in cold water and is known to produce a consistent increase in AP. Interestingly, the test does not evoke large increases in HR and cardiac output. Since it is a non‐invasive methodology, it is an interesting tool for assessing vascular function, mainly because evidence points to a vasomotor similarity between the carotid artery reactivity (CAR) and the reactivity of the coronary arteries (Peace et al., 2018; Van Mil et al., 2017). The CAR during CPT has shown good prognostic value for the development of cardiovascular disease or event (Peace et al., 2018). Evidence indicates that stimulation of SNS activity by CPT promotes vasodilation of the common carotid artery in healthy young adults. On the other hand, individuals at risk for cardiovascular disease may present attenuated vasodilation or even vasoconstriction in the common carotid artery in response to CPT (Peace et al., 2018; Pouwels et al., 2019; Van Mil et al., 2017).

The Stroop color‐word test (Stroop), in turn, is a neuropsychological instrument, proposed in 1935 (Stroop, 1935), designed to evaluate selective attention processing. The Stroop has also been shown to induce acute mental stress, evoking hemodynamic and autonomic responses (Barbosa et al., 2010; Gauche et al., 2017). Studies from our research group (Formolo et al., 2022; Mendes et al., 2024) have shown that the Stroop increases HR and AP, mainly systolic AP (SAP), in different populations such as healthy individuals, individuals with chronic diseases (i.e., obesity), and firefighters. Nevertheless, the Stroop effects on vascular reactivity are not fully established. A single study has evaluated CAR to mental stress (Naqvi & Hyuhn, 2009). The study findings indicate vasodilation of the common carotid artery in healthy participants and attenuation of this response in hypertensive individuals. However, the authors used three stressful stimuli, including Stroop, and determined the CAR as the largest response among the stimuli. Furthermore, the CAR was assessed only at the end, and not during each stimulus.

Given the above, further studies are needed to better understand the CAR to mental stress induced by Stroop. Since Stroop‐induced mental stress and CPT‐induced physical stress stimulate the SNS, we hypothesized that mental stress triggers a significant CAR, comparable to that observed in response to cold. Thus, the study aimed to compare common carotid artery hemodynamics and autonomic modulation reactivity induced by mental stress and cold.

## 2 MATERIALS AND METHODS

### 2.1 Participants and ethical aspects

The study included males and females aged between 18 and 40 years. The exclusion criteria were: (I) presence of cardiovascular, mental, or metabolic diseases, or infectious or inflammatory processes; (II) smokers; (III) people using drugs that could influence the variables analyzed; (IV) waist‐to‐height ratio (WHtR) >0.5; (V) athletes (training for performance in amateur or professional competitions) or insufficiently active individuals (<150 min/week of moderate/vigorous physical activity), assessed by the International Physical Activity Questionnaire (IPAQ).

Participants were recruited through social media, posters, and institutional e‐mails. The sample size was calculated considering type I (*α* = 0.05) and type II (*β* = 0.20) errors to identify differences of at least 0.25 (small effect) in the CAR (paired Student's *t*‐test) as previously observed in the CAR to CPT by our research group (Teixeira et al., 2023). The G*Power software version 3.1.9.2 (Faul et al., 2007) was used to calculate the sample size, generating a result of 20 participants. Considering a loss rate of 10%, the final estimated sample size was up to 22 participants. The research was approved by the Human Research Ethics Committee of the Federal University of Santa Catarina (UFSC) (no. 68282923.1.0000.0121) and conducted in accordance with the Declaration of Helsinki. All participants signed the free and informed consent form. The Consolidated Standards of Reporting Trials (CONSORT) guidelines were used to report the study (Dwan et al., 2019; Moher et al., 2010). The study was registered in the Brazilian Registry of Clinical Trials (ReBEC; RBR‐4hrvmkb).

### 2.2 Experimental design

**FIGURE 1:**
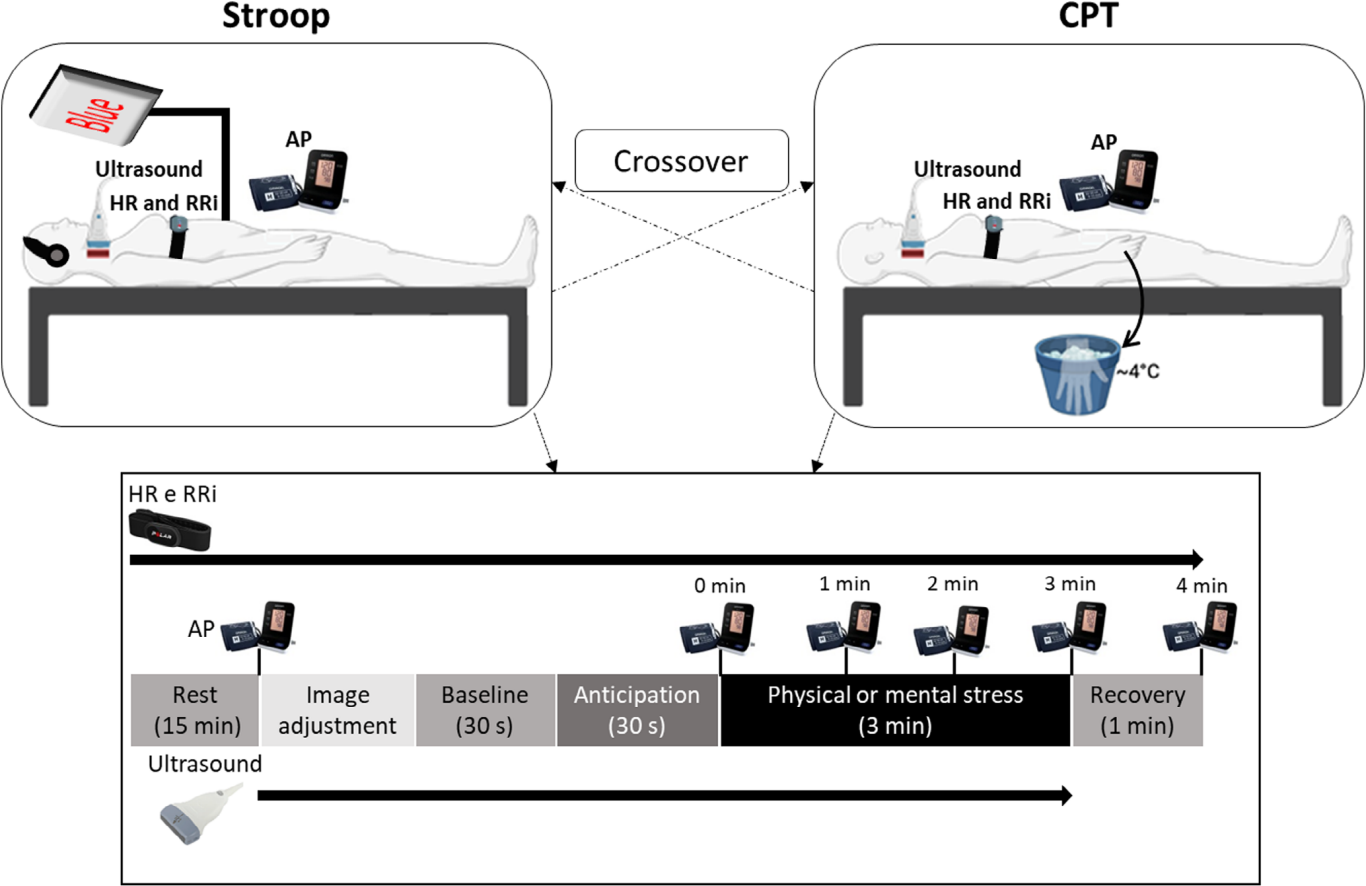
Experimental design. Arterial pressure (AP); cardiac intervals (RRi); cold pressor test (CPT); heart rate (HR); Stroop color‐word test (Stroop).

This was a randomized crossover experimental study. Participants were assessed during a single visit to the Cardiac & Vascular Physiology Laboratory (CardioVasc Lab) at the UFSC, between October 2023 and June 2024. Assessments were conducted in the morning, with at least a 6 h fast before eating, 24 h without physical activity, and abstention from alcoholic and caffeinated beverages 12 h before the study. This standardization was intended to minimize the influence of these variables on the outcomes of interest. Females were assessed between the second and sixth day of the hormonal cycle (self‐reported) to avoid the impact of hormonal fluctuations on the outcomes assessed. When the participants arrived at the laboratory, they signed the informed consent form, answered the IPAQ, filled out a document with their sociodemographic data, and performed anthropometric measurements (body mass, height, and waist circumference). Participants then rested for 15 min in the supine position. Subsequently, participants completed the CPT and Stroop in a randomized order, each lasting 3 min, with a 15‐min interval between them. Ultrasound of the common carotid artery was performed 1 min before (30 s baseline and 30 s anticipation) and during the tests to assess CAR variables. AP was measured at the end of the rest period, immediately before (anticipation), during (at the end of each minute), and after (1‐min recovery) the tests. HR and R‐R intervals (RRi) were measured continuously throughout the role experiment. Immediately after each test, the participants reported their perceived stress using the following scores: 0 = not stressful, 1 = slightly stressful, 2 = stressful, 3 = very stressful, and 4 = extremely stressful (Gauche et al., 2017). The experimental design is shown in Figure 1.

### 2.3 Randomization and data analysis

We used simple randomization, and the order of interventions for each participant was generated by random allocation sequence generation software (Microsoft Excel®, Microsoft®, Redmond, WA, USA). The assessment of the primary outcome (CAR) was conducted by an experienced researcher, and the analysis was performed by an experienced, blinded researcher.

### 2.4 Cold pressor test

The CPT protocol was conducted in accordance with a previous study (Peace et al., 2020). Briefly, participants lay in a supine position, positioned close to the right edge of the bed to ensure that their hand could move in the water without significant neck movement. They submerged the right hand in cold water (~4°C) for 3 min and were instructed to breathe normally and not to talk during the tests.

### 2.5 Stroop color‐word test

Mental stress was induced using a computerized version of the Stroop adapted from previous studies (Formolo et al., 2022; Gauche et al., 2017). The procedure involved the presentation of automated slides on a monitor placed in front of participants lying in the supine position. Before starting the Stroop rest period, the participants underwent a familiarization with the test, which included an explanation and a 15‐s simulation of the test. Each slide lasted 1 s and showed the names of colors (i.e., blue, yellow, and red) in colors different from those indicated by the name, where each participant used a numeric keypad to indicate the color of the letters of the word as quickly as possible, rather than the name of the written word. Three possible color options were associated with the numeric keypad. In addition, there was an auditory conflict (a recorded voice) saying the names of different colors in a headset that was provided during the test to increase confounding factors.

### 2.6 Carotid artery reactivity

The CAR analysis was based on previous studies (Peace et al., 2020; Teixeira et al., 2023). The diameter of the right common carotid artery was assessed by high‐resolution ultrasonography (GE Versana, General Electric Medical Systems, Milwaukee, WI, USA). Briefly, the common carotid artery (proximal to the carotid bulb) was identified, and the image was optimized to ensure a good definition of the artery walls. Doppler velocity was also recorded at the smallest possible insonation angle (always <60°). The diameter of the common carotid artery was calculated using edge detection software (Cardiovascular Suite, Quipu, Pisa, Italy). The baseline was calculated from the average of the first 30 s of recording. CAR was assessed using the moving average of 10 s windows. The 30 s before the start of the interventions were included in the CAR due to the possible anticipatory response to the stimuli. From this moving average, the area under the curve (AUC), peak diameter, time to peak, maximum reactivity (mm), maximum reactivity (%), and the effective response time (ERT) were calculated. The classification of the CAR was based on the AUC; when the AUC was positive, the maximum positive reactivity was calculated, and when the AUC was negative, the maximum negative reactivity was calculated. ERT was defined as a CAR above 1.5% and below −1.5% (Vermeulen et al., 2022).

### 2.7 Arterial pressure

SAP and diastolic AP (DAP) were measured using an automated oscillometric device (Omron Healthcare, Kyoto, Japan) with the participant in the supine position, with the left arm supported and relaxed. The mean AP (MAP) was calculated as 1/3 SAP +2/3 DAP. AP data are presented in mmHg.

### 2.8 Heart rate and autonomic modulation to the heart

HR and RRi were recorded using the Polar H10 HR sensor and the Elite HRV smartphone app (Formolo et al., 2022). Time series of spontaneously breathing RRi were used to assess HRV, which infers autonomic modulation to the heart. The square root of the mean square of the differences between adjacent normal RRi (RMSSD) and standard deviation of NN intervals (SDNN) were evaluated in the time domain, and the high frequency (HF; 0.15–0.4 Hz) was evaluated in the frequency domain, using the Kubios HRV Standard software (Kubios Oy, Kuopio, Finland). SDNN infers total variability while RMSSD and HF infer the parasympathetic modulation of the heart (Task Force, 1996). To capture subtle changes in autonomic reactivity to the CPT or Stroop, we analyzed ultra‐short HRV using 1‐min RRi time series before, during, and after the tests, as previously described (Esco & Flatt, 2014; Salahuddin et al., 2007; Teixeira et al., 2023; Volpes et al., 2022). The time series were examined, and the removal of ectopic beats and artifacts was performed using filters, considering a maximum acceptable loss of 5% of beats. SDNN and RMSSD are presented in ms and HF in natural logarithm (Ln) ms^2^.

### 2.9 Statistical analysis

Statistical analysis was performed using GraphPad Prism 6 software (Graphpad Inc., La Jolla, USA). Initially, the Shapiro–Wilk test was applied to the data to assess data normality. For descriptive statistics, data were expressed as mean ± standard deviation (SD) for parametric data and median (percentiles p25– p75) for nonparametric data. In the Tables, CAR variables were presented in absolute and relative (percentage and delta) values while hemodynamic and autonomic data were presented in maximum (delta peak) reactivity. In the Graphs, CAR and hemodynamic and autonomic data were presented in relative response.

For the inferential statistics, paired Student's *t*‐test (parametric data) or Wilcoxon (nonparametric data) was used for comparisons between two means or medians. A two‐way analysis of variance (ANOVA) for repeated measures, followed by Sidak's post‐hoc test, was used to compare continuous variables over time between the two interventions. Significance level was set at *α* = 0.05.

Effect size was calculated using means and standard deviations using Cohen's d_z_ for paired samples for parametric data and rank‐biserial correlation for nonparametric data, considering small (0.20 ≤ d < 0.50), medium (0.50 ≤ d < 0.80), and large (d ≥ 0.80) effects (Cohen, 1988; Lakens, 2013).

## 3 RESULTS

### 3.1 Participants

**FIGURE 2:**
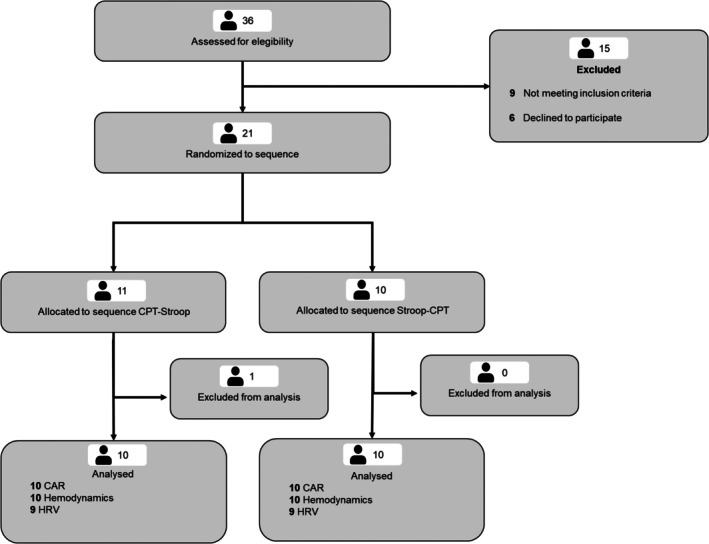
Consolidated Standards of Reporting Trials (CONSORT) flow diagram for crossover trials. Cold pressor test (CPT); Stroop color‐word test (Stroop). Carotid artery reactivity (CAR); Heart rate variability (HRV).

Eligibility criteria were assessed in 36 potential participants. Nine individuals did not meet the inclusion criteria, and six declined to participate for personal reasons. One participant was excluded because, after randomization and assessment, we identified that she met one of the exclusion criteria (WHtR >0.5). Therefore, 20 participants were included in the final analysis (10 females and 10 males). Two participants were excluded from the HRV analysis due to recording errors. The participant flow is presented in Figure 2.

### 3.2 Characterization of participants

Table 1 presents the data characterizing the participants.

**TABLE 1:** Characterization of participants.

	Stroop‐CPT (*n* = 10)	CPT‐Stroop (*n* = 10)	Total (*n* = 20)
Females (*n*)	5	5	10
Age (years)	25.2 ± 3.7	26.3 ± 6.5	25.7 ± 5.2
Height (m)	1.76 ± 0.10	1.68 ± 0.10	1.72 ± 0.10
Body mass (Kg)	69.1 (61.6–76.3)	65.5 (63.5–68.9)	65.7 (62.7–72.8)
BMI (Kg/m^2^)	22.9 (21.9–23.8)	23.3 (22.2–24.6)	22.9 (22.1–24.4)
WC (cm)	71.5 (70.1–80.5)	72.3 (71.6–75.9)	72.0 (70.4–78.0)
WHtR	0.42 ± 0.03	0.44 ± 0.03	0.43 ± 0.03
SAP (mmHg)	112.7 ± 13.8	114.9 ± 10.8	113.9 ± 11.5
DAP (mmHg)	67.9 ± 8.1	67.9 ± 5.6	67.4 ± 5.3
MAP (mmHg)	82.3 ± 9.4	83.5 ± 7.0	82.9 ± 6.8
HR (bpm)	57.5 ± 10.2	66.0 ± 9.5	61.2 ± 9.1
MVPA (min/week)	245 (180–440)	200 (150–300)	230 (170–360)

*Note*: Values presented as mean ± standard deviation for parametric data and median (percentiles p25–p75) for nonparametric data.

Abbreviations: BMI, body mass index; CPT, cold pressure test; DAP, diastolic arterial pressure; HR, heart rate; MAP, mean arterial pressure; MVPA, moderate and vigorous physical activity; SAP, systolic arterial pressure; WC, waist circumference; WHtR, waist‐to‐height ratio.

### 3.3 Carotid artery reactivity to Stroop and CPT

**FIGURE 3:**
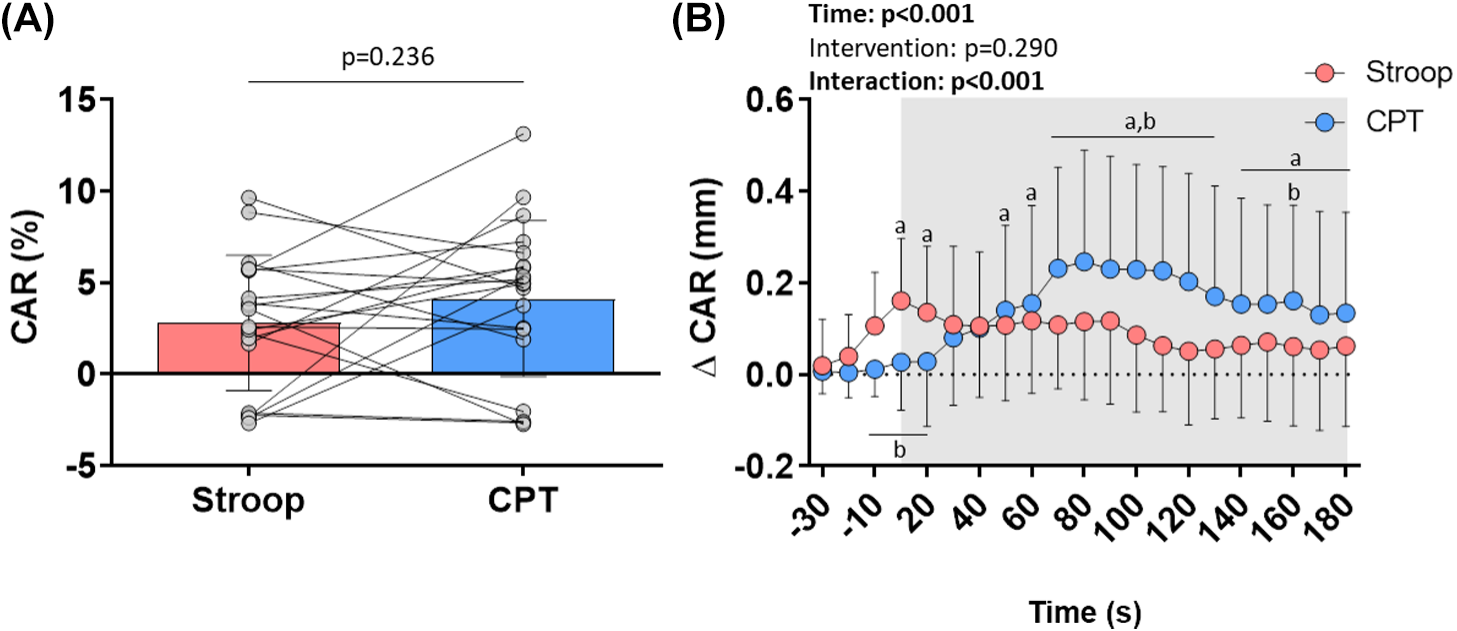
Carotid artery reactivity (CAR) to the Stroop color‐word test (Stroop) and cold pressor test (CPT). Values are presented as mean ± standard deviation. Paired Student's *t*‐test (panel A) and two‐way repeated‐measures ANOVA with Sidak's post‐hoc test (panel B); *p* < 0.05; ^a^significant vs. –30s (baseline); ^b^significant vs. Stroop; *n* = 20. The gray highlight in panel B represents the period in which participants performed the Stroop or CPT.

The CAR, assessed by the maximum response percentage (dilation or constriction) from 10‐s averages throughout the test, was similar in Stroop and CPT (Figure 3A and Table 2). Repeated‐measures ANOVA analysis for CAR as a function of time indicated a time × intervention interaction and time effect. Post‐hoc analysis revealed an increase in the diameter of the common carotid artery during Stroop between 10 and 20 s and in CPT between 50 and 180 s compared to the baseline diameter. Furthermore, comparing the two interventions, the diameter of the common carotid artery was larger in Stroop between −10 and 20 s, and larger in CPT between 50 and 130 s and 160 min (Figure 3B, Table S1).

**TABLE 2:** Carotid artery reactivity to Stroop and CPT.

	Stroop	CPT	*p*‐value	Effect size
Baseline diameter (mm)	6.5 ± 0.3	6.5 ± 0.2	0.133 ^t^	0.00^d^
Peak diameter (mm)	6.7 ± 0.3	6.8 ± 0.2	**0.046** ^**t**^	0.39^d^
CAR (%)	2.8 ± 3.6	4.1 ± 4.2	0.236^t^	0.33^d^
AUC (cm s)	1.7 ± 2.8	2.8 ± 3.3	0.278^t^	0.36^d^
Time to peak (s)	66.5 ± 56.5	99.5 ± 35.1	**0.020** ^**t**^	0.70^d^
ERT (s)	60.0 (32.5–157.5)	115.0 (42.5–157.5)	0.298^w^	0.28^r^

*Note*: Values presented as mean ± standard deviation for parametric data and median (percentiles p25–p75) for nonparametric data; ^t^Paired Student's *t*‐test or ^w^Wilcoxon; ^d^Cohen's d or ^r^rank‐biserial correlation; *p* < 0.05; *n* = 20. Bold values indicate statistically significant comparisons.

Abbreviations: AUC, area under the curve; CAR, carotid artery reactivity; ERT, effective response time.

The baseline diameter, AUC, and effective response time (ERT; >1.5% or <−1.5%) were similar between the interventions. The time to peak was shorter in Stroop (medium effect size), while the peak diameter was larger in CPT (small effect size; Table 2).

### 3.4 Hemodynamic reactivity to Stroop and CPT

**FIGURE 4:**
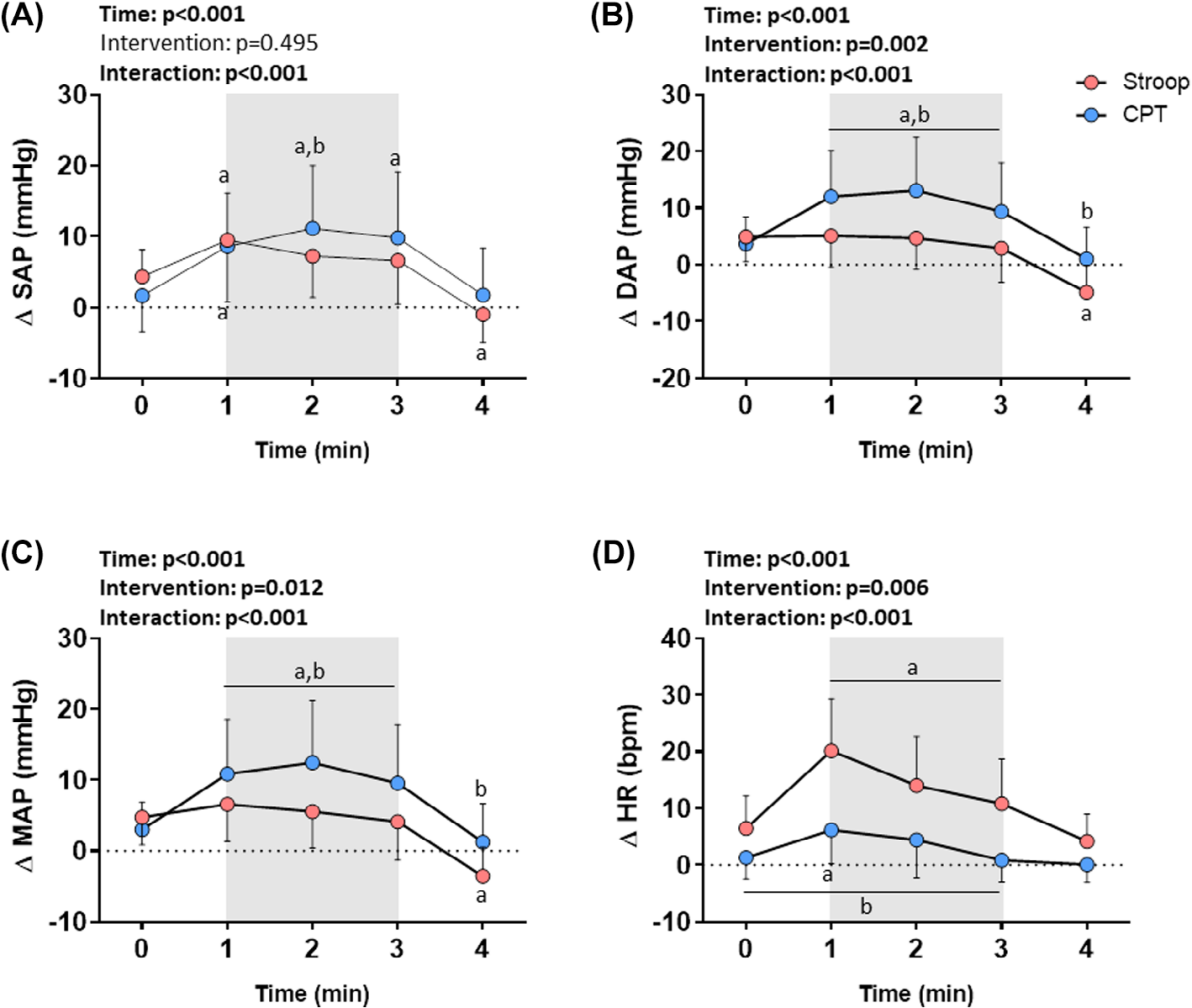
Reactivity of (A) systolic (SAP), (B) diastolic (DAP), and (C) mean (MAP) arterial pressure, and (D) heart rate (HR) to the Stroop color‐word test (Stroop) and cold pressor test (CPT). Values are presented as mean ± standard deviation. ANOVA for repeated measures followed by Sidak's post‐hoc test; *p* < 0.05; ^a^significant vs. rest (0′); ^b^significant vs. Stroop; *n* = 20. The gray highlight in the panels represents the period in which the participants performed the Stroop or CPT.

The hemodynamic data are presented in Figure 4. Repeated‐measures ANOVA indicated an effect of time and time × intervention interaction for SAP, DAP, MAP, and HR, as well as an intervention effect for DAP, MAP, and HR. Post‐hoc analysis for SAP indicated an increase at the 1^st^ min and a decrease in the recovery in Stroop, and an increase from the 1^st^ min to the 3^rd^ min in CPT compared to baseline. SAP was higher in the second min in CPT compared to Stroop (Figure 4A, Table S2). For DAP, there was a decrease in the recovery in Stroop and an increase from the 1^st^ to the 3^rd^ min in CPT compared to baseline. DAP was higher from the 1^st^ to the 3^rd^ min of the test and during recovery in CPT compared to Stroop (Figure 4B, Table S3). For MAP, there was an increase from the 1^st^ to the 3^rd^ min in CPT and a reduction in the recovery in Stroop compared to baseline. MAP was higher from the 1^st^ to the 3^rd^ min of the test and during recovery in CPT compared to Stroop (Figure 4C, Table S4). For HR, there was an increase from the 1^st^ to the 3^rd^ min in Stroop and in the 1^st^ min in CPT. HR was higher from baseline to the 3rd min of the test and in Stroop compared to CPT (Figure 4D. Table S5).

Regarding the maximum hemodynamic responses, the t or Wilcoxon test indicated greater DAP and MAP responses in CPT (large and medium effect sizes, respectively) and a higher HR response in Stroop (medium effect size), while the maximal SAP response was similar in both interventions (Table 3).

**TABLE 3:** Maximum hemodynamic reactivity to the Stroop and CPT.

	Stroop	CPT	*p*‐value	Effect size
∆ SAP (mmHg)	10.6 ± 6.4	12.8 ± 9.2	0.380^t^	0.28^d^
∆ DAP (mmHg)	8.5 (6.2–10.0)	13.0 (6.2–23.5)	**0.009** ^**w**^	0.96^r^
∆ MAP (mmHg)	8.4 ± 4.2	13.5 ± 8.5	**0.023** ^**t**^	0.76^d^
∆ HR (bpm)	20.5 (10.2–27.7)	11.0 (5.2–14.7)	**0.013** ^**w**^	0.61^r^

*Note*: Values are presented as mean ± standard deviation for parametric data and median (percentiles p25–p75) for nonparametric data; ^t^Paired Student's *t*‐test or ^w^Wilcoxon; ^d^Cohen's d or ^r^rank‐biserial correlation; *p* < 0.05; *n* = 20. Bold values indicate statistically significant comparisons.

Abbreviations: CPT, cold pressor test; DAP, diastolic arterial pressure; HR, heart rate; MAP, mean arterial pressure; SAP, systolic arterial pressure; Stroop, Stroop color‐word test.

### 3.5 HRV reactivity to Stroop and CPT

**FIGURE 5:**
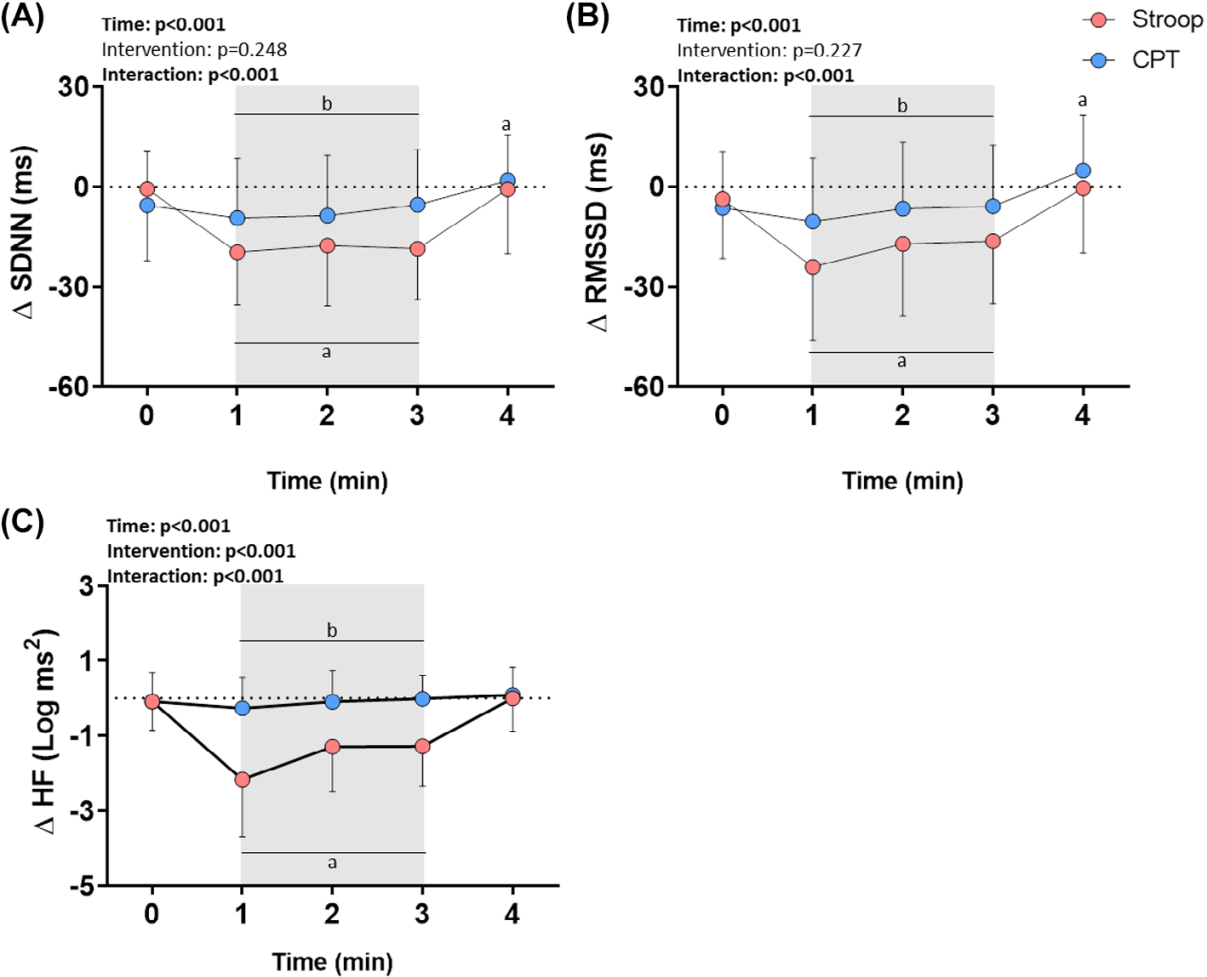
Reactivity of (A) Standard deviation of NN intervals (SDNN); (B) Root mean square of the sum of the squared difference between adjacent R‐R intervals (RMSSD), and (C) High frequency (HF) to the Stroop color‐word test (Stroop) and cold pressor test (CPT). Values are presented as mean ± standard deviation. ANOVA for repeated measures followed by Sidak's post‐hoc test; *p* < 0.05; ^a^significant vs. rest (0′); ^b^significant vs. Stroop; *n* = 18. The gray highlight in the panels represents the period in which the participants performed the Stroop or CPT.

Repeated‐measures ANOVA revealed an effect of time and time × intervention interaction for SDNN, RMSSD, and HF, in addition to an effect of intervention for RMSSD. Post‐hoc analysis of SDNN, RMSSD, and HF indicated a reduction from the 1^st^ to the 3^rd^ min in Stroop. During recovery, the RMSSD increased in the CPT. All variables were lower from the 1^st^ to the 3^rd^ min of Stroop compared to CPT (Figure 5, Tables S6–S8).

The maximum responses confirmed these results, indicating lower SDNN, RMSSD, and HF (medium, medium, and large effect sizes, respectively) in Stroop (Table 4).

**TABLE 4:** Maximum HRV reactivity to the Stroop and CPT.

	Stroop	CPT	*p*‐value	Effect size
∆ SDNN (ms)	−20.2 (−30.3 to −13.8)	−15.1 (−23.0 to −8.2)	**0.043^w^**	0.54^r^
∆ RMSSD (ms)	−21.3 (−39.6 to −10.0)	−12.8 (−22.8–0.95)	**0.026^w^**	0.59^r^
∆ HF (Log ms^2^)	−2.3 ± 1.3	−0.61 ± 0.74	**<0.001^t^**	1.60^d^

*Note*: Values are presented as mean ± SD for parametric data and median (quartiles p25–p75) for nonparametric data. ^t^Paired Student's *t*‐test or ^w^Wilcoxon; ^d^Cohen's d or ^r^rank‐biserial correlation; *p* < 0.05; *n* = 18. Bold values indicate statistically significant comparisons.

Abbreviations: CPT, cold pressor test; HF, high frequency; RMSSD, root mean square of the sum of the squared difference between adjacent R‐R intervals; SDNN, standard deviation of NN intervals; Stroop, Stroop color‐word test.

We also assessed the perceived stress during Stroop and CPT. Results indicated higher perceived stress during the CPT compared to Stroop [2.5 (2–3) vs. 2 (1–2) arbitrary units; *p* = 0.034; medium effect size: 0.60; not shown].

## 4 DISCUSSION

This study aimed to compare the CAR induced by mental or cold stress. We also evaluated similarities and differences between mental and cold stress in hemodynamic reactivity and autonomic modulation of the heart. Since mental stress induced by Stroop or cold stress induced by CPT stimulates the SNS and evokes AP responses (Formolo et al., 2022; Mendes et al., 2024; Peace et al., 2020; Van Mil et al., 2017), we hypothesized that Stroop would be able to evoke a comparable CAR to that caused by CPT. The hypothesis of the present study was confirmed, as the percentage of CAR reactivity was similar between the Stroop and CPT, despite differences in their time courses. Regarding hemodynamic and autonomic modulation responses, CPT promoted a higher increase in DAP and MAP, while Stroop demonstrated a greater increase in HR and a higher reduction in parasympathetic modulation to the heart.

### 4.1 Carotid artery reactivity to Stroop and CPT

In our study, we observed no differences between the magnitude of CAR to Stroop and CPT. Specifically, we found a similar response in percentage, the AUC, and the TRE (>1.5% or <−1.5%) between interventions. However, the time to peak was shorter in the Stroop, and there was a significant time × intervention interaction in the time course analysis.

The CPT has been used for many years as an acute stress test to assess the sympathetic neural influence on peripheral circulation and coronary circulation in humans (Schächinger et al., 2000; Velasco et al., 1997). Furthermore, there are several studies in scientific literature evaluating CAR to CPT (Peace et al., 2020; Van Mil et al., 2017; van Mil et al., 2019). As observed in our findings, previous studies indicate that CPT promotes vasodilation of the common carotid artery in healthy individuals (Peace et al., 2018, 2020). Nevertheless, individuals with or at risk for cardiovascular disease may exhibit vasoconstriction of the common carotid artery in response to the CPT (Peace et al., 2018; Pouwels et al., 2019; Van Mil et al., 2017).

Regarding mental stress, we found only one study in the literature that evaluated the CAR, indicating vasodilation of the common carotid artery in healthy participants and attenuation of this response in hypertensive individuals (Naqvi & Hyuhn, 2009). However, the interpretation of the findings of this previous study is limited and not very specific, since the authors used three stressful stimuli (Stroop, descending subtraction task, and anger recall test) and determined the CAR as the greatest response among these stimuli. In addition, the CAR was only verified after the tests were completed. Our study evaluated the temporal course of the CAR in response to a specific mental stressor, as well as the comparison with an already consolidated test (i.e., CPT). We understand that our approach favors a more robust and comprehensive analysis.

Although the magnitude of the CAR response percentage was similar between the two types of stress, it is noteworthy that the time until the peak occurred earlier in mental stress compared to physical stress. Furthermore, our findings show that, on average, there was an increase in the common carotid artery diameter at the onset of the Stroop, which was not observed in the CPT. This response is consistent with the anticipation of physiological responses to standardized tests involving mental stress observed in previous studies (Shilton et al., 2017).

Our experimental protocol does not allow us to indicate the specific mechanisms involved in the CAR in the Stroop. We hypothesize that SNS activation evoked by mental stress stimulates adrenal epinephrine release, leading to carotid vasodilation through the activation of β2‐adrenergic receptors (Peace et al., 2018; Pouwels et al., 2019). Future studies should test this hypothesis.

### 4.2 Hemodynamic and autonomic reactivity to Stroop and CPT

It has been demonstrated that Stroop is a useful and efficient tool to evoke hemodynamic responses (Barbosa et al., 2010; Fauvel et al., 1996; Gauche et al., 2017). Recent studies by our research group have demonstrated that acute mental stress induced by Stroop is capable of increasing HR and AP, mainly SAP, in different populations such as healthy individuals, firefighters, and people with obesity (Formolo et al., 2022; Mendes et al., 2024). The findings of the present study agree with these previous studies since we observed a considerable hemodynamic response using similar BP monitoring methods, with an increase, mainly, in SAP and HR. It is worth mentioning that, in the present study, Stroop was performed in the supine position, while in previous studies the test was performed in the sitting position. This adaptation was made to allow the CAR analysis during the Stroop. Despite this, the Stroop induced significant hemodynamic and autonomic reactivity. Furthermore, peak hemodynamic reactivity occurred at the beginning of the Stroop, again indicating an anticipation of the physiological response to mental stress.

The CPT is also widely used to evaluate the hemodynamic response, leading mainly to an increase in AP (Hines & Brown, 1936). As expected, in the present study we found significant AP reactivity. However, unlike the Stroop, cold‐induced stress indicated a marked increase in DAP throughout the test. A previous study showed that the SNS stimulation induced by the CPT contributes mainly to the release of norepinephrine, which contrasts with the release of norepinephrine and epinephrine in mental stressor (Sherwood et al., 1995), contributing to peripheral vasoconstriction (Velasco et al., 1997). Also, CPT involves an important reduction in skin temperature, which may lead to a sympathetic vasoconstriction thermoregulatory response. In some cases, local cooling led to a decrease in endothelial nitric oxide synthase activity, also contributing to vasoconstriction (Alba et al., 2019). Altogether, these mechanisms might explain the increased total peripheral resistance in CPT compared to mental stressors observed in previous studies (Sherwood et al., 1986, 1995), and why DAP increase is greater in CPT than in Stroop.

The peripheral vasoconstriction and AP increase induced by CPT may activate a baroreflex response, which may explain the attenuated increase in HR compared to Stroop, and even a tendency to return to baseline levels throughout the test. A previous study (Mourot et al., 2009) had already demonstrated a CPT‐induced small increase in HR in the 1st min of the test, with this variable returning to baseline levels in the 2nd min. Paradoxically, the authors also demonstrated increases in muscular SNS activity in the 1st min, with its peak occurring in the 2nd min. According to the authors, a possible interpretation of these findings is that the decrease in HR during CPT is related to a baroreflex response evoked by the sustained increase in AP. In this situation, there may be coactivation of both arms of the ANS, leading to a decrease in HR or limiting HR increase to the sympathetic stimulus (Carrive, 2006; Mourot et al., 2009). Indeed, our data on autonomic modulation reactivity, inferred by the HRV, do not indicate a significant reduction in the vagal modulation during the CPT. On the contrary, we observed a reduction in the parasympathetic modulation during the Stroop, corroborating the robust increase in HR. Our research group had already demonstrated, using HRV, a significant decrease in the parasympathetic modulation to the heart during acute mental stress induced by the Stroop (Formolo et al., 2022; Mendes et al., 2024), but not during the CPT (Teixeira et al., 2023).

### 4.3 Specificities of mental and physical stress

Both mental and physical stressors activate homeostatic systems to adapt, producing compensatory responses that help to cope with stress (Chrousos, 2009). Both types of stress involve the activation of the HPA axis and the SNS, resulting in the release of hormones such as cortisol and adrenaline (Godoy et al., 2018; Lundberg, 2006). Interestingly, in stressors involving mental stress, there is an anticipation of stress, that is, an activation of response systems, preparing the organism even before the onset of the stressful event (Shilton et al., 2017). Although the neural response to CPT also encompasses cortical processing in response to the thermal stimuli, it seems that CPT responses are also mediated by reflexive mechanisms (Lovallo, 1975). Our data on hemodynamic and carotid reactivity to the Stroop and CPT corroborate this thesis since we observed anticipation in mental stress. Also, it is worth noting that five participants did not show consistency in the CAR on the Stroop and CPT, with a vasodilation profile for one test and vasoconstriction for the other. This finding may be related to the specificity of the pathways activated by the two types of stress and/or the individuality of the participants in response to them.

This scenario may reflect processes of response planning, which rely on attention and memory (Schulkin et al., 1994). Cognitive appraisal prior to a stressor has been shown to shape the endocrine response (Gaab et al., 2005), supporting the notion that anticipatory evaluation can directly influence physiological outcomes. Unlike the passive nature of the CPT, the Stroop task includes elements of social evaluation and performance motivation, both of which have been associated with stronger endocrine responses (Dickerson & Kemeny, 2004). These factors may also contribute to the distinct patterns of cardiovascular reactivity observed across stress paradigms.

The specific pathways involved in each stress type help to explain, at least in part, our findings. The response to mental stress depends on complex cognitive processes involving brain regions such as the prefrontal cortex and the amygdala, crucial areas for processing emotional and cognitive information. The interaction between these areas plays a fundamental role in emotional regulation and activation of the autonomic response (Kern et al., 2008; Russo & Nestler, 2013; Skoluda et al., 2015; Ulrich‐Lai & Herman, 2009). On the other hand, the response to physical stress, such as that induced by CPT, seems to have a greater contribution of areas such as the brainstem and the hypothalamus, evoking rapid systemic reactions (Godoy et al., 2018). Activation of the sympathetic adrenomedullary system increases vigilance and alertness (Joëls & Baram, 2009; Ulrich‐Lai & Herman, 2009).

### 4.4 Limitations of the study

The study's limitations include the lack of beat‐to‐beat AP measurement, which would allow a more detailed assessment of this variable besides the analysis of SNS modulation to the vessels, which could help to interpret our CAR and hemodynamic findings. Furthermore, we recognize that the lack of beat‐to‐beat AP might also have limited the temporal profile for AP reactivity, since a single AP measurement might represent the peak response. The sample size did not allow for specific analyses and comparisons related to sex. Finally, we did not collect data related to ethnicity or symptoms of stress, anxiety, and depression, factors that may influence the outcomes assessed.

## 5 CONCLUSION

The findings of the present study indicate that the CAR evoked by acute mental stress induced by the Stroop has a magnitude similar to that provoked by physical stress induced by the CPT test. However, the CAR to mental stress is evoked at the onset of the stressful stimulus and reaches its peak earlier when compared to physical stress. These findings align with the typical hemodynamic response profile to both types of stress, suggesting an anticipatory reaction to mental stress that may be associated with the activation of distinct brain networks specific to each stressor. Furthermore, the present study suggests that the CAR to mental stress induced by the Stroop has the potential to be used to assess vascular function. Although both are simple and low‐cost tests, the Stroop seems to generate a lower perception of stress when compared to the CPT test. In addition, the CPT may evoke pain and discomfort in people who are overly sensitive to cold or even tissue damage in cases such as Raynaud's syndrome. Future studies in people with chronic diseases and increased risk of developing them are necessary to elucidate this potential and analyze the CAR as a predictor of cardiovascular events.

## AUTHOR CONTRIBUTIONS

G.F.S. and F.J.F. conceived and designed the study. F.J.F., I.G.T., W.O., R.F., and F.S. conducted the experiments. F.J.F., I.G.T., G.F.S, W.O. and F.S. analyzed the data. G.F.S, R.F., and F.J.F. prepared the initial draft of the manuscript. All authors revised the manuscript critically for important intellectual content, approved the final version of the manuscript and agreed to be accountable for all aspects of the work.

## FUNDING INFORMATION

This work was supported by Master's and Ph.D. scholarships from the Conselho Nacional de Desenvolvimento Científico e Tecnológico (CNPq) and the Coordenação de Aperfeiçoamento de Pessoal de Nível Superior (CAPES). Guilherme F. Speretta acknowledges support from a CNPq Research Productivity Fellowship (Project No. 307997/2025‐1).

## CONFLICT OF INTEREST STATEMENT

The authors declare no conflict of interest.

## Supporting information


**Table S1.** Carotid artery reactivity (CAR) to Stroop and cold pressor test (CPT) over time.
**Table S2.** Systolic arterial pressure reactivity to Stroop and cold pressor test (CPT over time).
**Table S3.** Diastolic arterial pressure (DAP) reactivity to Stroop and cold pressor test (CPT over time).
**Table S4.** Mean arterial pressure (MAP) reactivity to Stroop and cold pressor test (CPT) over time.
**Table S5.** Heart rate (HR) reactivity to Stroop and cold pressor test (CPT) over time.
**Table S6.** Standard deviation of NN intervals (SDNN) reactivity to Stroop and cold pressor test (CPT) over time.
**Table S7.** Root mean square of the sum of the squared difference between adjacent R‐R intervals (RMSSD) reactivity to Stroop and cold pressor test (CPT) over time.
**Table S8.** High Frequency (HF) reactivity to Stroop and cold pressor test (CPT) over time.

## Data Availability

The raw data will be made available by the authors upon reasonable request.
