# Supplementary material for: Similarities and differences in carotid artery, hemodynamic, and autonomic reactivity induced by mental stress or cold in adults: A randomized crossover study
Source: Physiol Rep. 2026 Mar 11;14(5):e70803. doi: 10.14814/phy2.70803 (PMC13097317; doi:10.14814/phy2.70803)
Supplement: Supplementary file 1 — Table S1. Carotid artery reactivity (CAR) to Stroop and cold pressor test (CPT) over time. Table S2. Systolic arterial pressure reactivity to Stroop and cold pressor test (CPT over time). Table S3. Diastolic arterial pressure (DAP) reactivity to Stroop and cold pressor test (CPT over time). Table S4. Mean arterial pressure (MAP) reactivity to Stroop and cold pressor test (CPT) over time. Table S5. Heart rate (HR) reactivity to Stroop and cold pressor test (CPT) over time. Table S6. Standard deviation of NN intervals (SDNN) reactivity to Stroop and cold pressor test (CPT) over time. Table S7. Root mean square of the sum of the squared difference between adjacent R‐R intervals (RMSSD) reactivity to Stroop and cold pressor test (CPT) over time. Table S8. High Frequency (HF) reactivity to Stroop and cold pressor test (CPT) over time. [file PHY2-14-e70803-s001.docx]

**SUPPLEMENTARY MATERIAL**

**Table S1 . Carotid artery reactivity (CAR) to Stroop and cold pressor test (CPT) over time**

|  | **∆ CAR (mm)** | | **Stroop vs.**  **-30 s** | **CPT vs.**  **-30 s** | **Stroop vs. CPT** |
| --- | --- | --- | --- | --- | --- |
| **Time (s)** | **Stroop** | **CPT** | **p-value** | **p-value** | **p-value** |
| **- 30** | 0.02 ± 0.10 | 0.00 ± 0.04 | - | - | >0.999 |
| **- 20** | 0.04 ± 0.09 | 0.00 ± 0.05 | 0.999 | >0.999 | 0.997 |
| **- 10** | 0.10 ± 0.11 | 0.01 ± 0.05 | 0.651 | >0.999 | **0.046** |
| **10** | 0.16 ± 0.13 | 0.02 ± 0.10 | **0.001** | >0.999 | **<0.001** |
| **20** | 0.13 ± 0.14 | 0.02 ± 0.14 | **0.037** | >0.999 | **0.011** |
| **30** | 0.10 ± 0.17 | 0.08 ± 0.14 | 0.561 | 0.974 | 0.999 |
| **40** | 0.10 ± 0.15 | 0.10 ± 0.16 | 0.670 | 0.392 | >0.999 |
| **50** | 0.10 ± 0.16 | 0.14 ± 0.18 | 0.600 | **0.003** | 0.999 |
| **60** | 0.11 ± 0.15 | 0.15 ± 0.21 | 0.273 | **<0.001** | 0.995 |
| **70** | 0.10 ± 0.13 | 0.23 ± 0.22 | 0.589 | **<0.001** | **0.001** |
| **80** | 0.11 ± 0.17 | 0.24 ± 0.24 | 0.338 | **<0.001** | **<0.001** |
| **90** | 0.11 ± 0.18 | 0.23 ± 0.24 | 0.289 | **<0.001** | **0.005** |
| **100** | 0.08 ± 0.16 | 0.22 ± 0.22 | 0.998 | **<0.001** | **<0.001** |
| **110** | 0.06 ± 0.14 | 0.22 ± 0.22 | >0.999 | **<0.001** | **<0.001** |
| **120** | 0.05 ± 0.16 | 0.20 ± 0.23 | >0.999 | **<0.001** | **<0.001** |
| **130** | 0.05 ± 0.15 | 0.17 ± 0.24 | >0.999 | **<0.001** | **0.004** |
| **140** | 0.06 ± 0.15 | 0.15 ± 0.23 | >0.999 | **<0.001** | 0.071 |
| **150** | 0.07 ± 0.17 | 0.15 ± 0.21 | >0.999 | **<0.001** | 0.149 |
| **160** | 0.06 ± 0.17 | 0.16 ± 0.20 | >0.999 | **<0.001** | **0.025** |
| **170** | 0.05 ± 0.17 | 0.13 ± 0.22 | >0.999 | **0.0143** | 0.233 |
| **180** | 0.06 ± 0.17 | 0.13 ± 0.22 | >0.999 | **0.0085** | 0.337 |

Values presented as mean ± standard deviation. Sidak post-hoc test p-values; p < 0.05; n = 20.

**Table S2. Systolic arterial pressure reactivity to Stroop and cold pressor test (CPT over time**

|  | **∆ SAP (mmHg)** | | **Stroop vs.**  **0 min** | **CPT vs.**  **0 min** | **Stroop vs. CPT** |
| --- | --- | --- | --- | --- | --- |
| **Time (min)** | **Stroop** | **CPT** | **p-value** | **p-value** | **p-value** |
| **0** | 4.3 ± 3.8 | 1.6 ± 5.0 | - | - | 0.170 |
| **1** | 9.5 ± 6.6 | 8.6 ± 7.7 | **0.001** | **<0.001** | 0.962 |
| **2** | 7.2 ± 5.8 | 11.1 ± 8.8 | 0.225 | **<0.001** | **0.014** |
| **3** | 6.6 ± 6.1 | 9.8 ± 9.2 | 0.567 | **<0.001** | 0.067 |
| **4** | -1.0 ± 3.9 | 1.7 ± 6.6 | **<0.001** | >0.999 | 0.156 |

Values presented as mean ± standard deviation Sidak post-hoc test p-values; p < 0.05; n = 20.

**Table S3. Diastolic arterial pressure (DAP) reactivity to Stroop and cold pressor test (CPT over time**

|  | **∆ DAP (mmHg)** | | **Stroop vs.**  **0 min** | **CPT vs.**  **0 min** | **Stroop vs. CPT** |
| --- | --- | --- | --- | --- | --- |
| **Time (min)** | **Stroop** | **CPT** | **p-value** | **p-value** | **p-value** |
| **0** | 4.9 ± 4.4 | 3.7 ± 4.7 | - | - | 0.821 |
| **1** | 5.1 ± 5.5 | 12.0 ± 8.1 | >0.999 | **<0.001** | **<0.001** |
| **2** | 4.7 ± 5.5 | 13.1 ± 9.3 | >0.999 | **<0.001** | **<0.001** |
| **3** | 2.9 ± 5.9 | 9.4 ± 8.6 | 0.591 | **<0.001** | **<0.001** |
| **4** | -4.8 ± 4.7 | 1.0 ± 5.6 | **<0.001** | 0.220 | **<0.001** |

Values presented as mean ± standard deviation. Sidak post-hoc test p-values; p < 0.05; n = 20.

**Table S4. Mean arterial pressure (MAP) reactivity to Stroop and cold pressor test (CPT) over time**

|  | **∆ MAP (mmHg)** | | **Stroop vs.**  **0 min** | **CPT vs.**  **0 min** | **Stroop vs. CPT** |
| --- | --- | --- | --- | --- | --- |
| **Time (min)** | **Stroop** | **CPT** | **p-value** | **p-value** | **p-value** |
| **0** | 4.7 ± 3.8 | 3.0 ± 3.8 | - | - | 0.485 |
| **1** | 6.5 ± 5.2 | 10.8 ± 7.6 | 0.681 | **<0.001** | **0.001** |
| **2** | 5.5 ± 5.1 | 12.4 ± 8.8 | 0.998 | **<0.001** | **<0.001** |
| **3** | 4.1 ± 5.4 | 9.5 ± 8.2 | 0.999 | **<0.001** | **<0.001** |
| **4** | -3.5 ± 4.0 | 1.2 ± 5.4 | **<0.001** | 0.714 | **<0.001** |

Values presented as mean ± standard deviation. Sidak post-hoc test p-values; p < 0.05; n = 20.

**Table S5. Heart rate (HR) reactivity to Stroop and cold pressor test (CPT) over time**

|  | **∆ HR (bpm)** | | **Stroop vs.**  **0 min** | **CPT vs.**  **0 min** | **Stroop vs. CPT** |
| --- | --- | --- | --- | --- | --- |
| **Time (min)** | **Stroop** | **CPT** | **p-value** | **p-value** | **p-value** |
| **0** | 6.4 ± 5.7 | 1.3 ± 3.7 | - | - | **<0.001** |
| **1** | 20.1 ± 9.1 | 6.2 ± 5.9 | **<0.001** | **<0.001** | **<0.001** |
| **2** | 14.0 ± 8.7 | 4.4 ± 6.7 | **<0.001** | 0.091 | **<0.001** |
| **3** | 10.8 ± 7.8 | 0.8 ± 3.8 | **0.004** | >0.999 | **<0.001** |
| **4** | 4.1 ± 4.7 | 0.9 ± 3.1 | 0.429 | 0.973 | **0.004** |

Values presented as mean ± standard deviation. Sidak post-hoc test p-values; p < 0.05; n = 20.

**Table S6. Standard deviation of NN intervals (SDNN) reactivity to Stroop and cold pressor test (CPT) over time**

|  | **∆ SDNN (ms)** | | **Stroop vs.**  **0 min** | **CPT vs.**  **0 min** | **Stroop vs. CPT** |
| --- | --- | --- | --- | --- | --- |
| **Time (min)** | **Stroop** | **CPT** | **p-value** | **p-value** | **p-value** |
| **0** | -0.6 ± 11.2 | -5.5 ± 16.6 | - | - | 0.101 |
| **1** | -19.6 ± 15.7 | -9.3 ± 17.9 | **<0.001** | 0.192 | **<0.001** |
| **2** | -17.6 ± 18.1 | -8.5 ± 18.0 | **<0.001** | 0.300 | **0.002** |
| **3** | -18.5 ± 15.2 | -5.3 ± 16.5 | **<0.001** | 0.960 | **<0.001** |
| **4** | -0.6 ± 19.3 | 1.9 ± 13.5 | 0.993 | **0.013** | 0.373 |

Values presented as mean ± standard deviation. Sidak post-hoc test p-values; p < 0.05; n = 18.

**Table S7. Root mean square of the sum of the squared difference between adjacent R-R intervals (RMSSD) reactivity to Stroop and cold pressor test (CPT) over time**

|  | **∆RMSSD (ms)** | | **Stroop vs.**  **0 min** | **CPT vs.**  **0 min** | **Stroop vs. CPT** |
| --- | --- | --- | --- | --- | --- |
| **Time (min)** | **Stroop** | **CPT** | **p-value** | **p-value** | **p-value** |
| **0** | -3.6 ± 14.0 | -6.3 ± 15.1 | - | - | 0.878 |
| **1** | -24.0 ± 22.0 | -10.3 ± 18.9 | **<0.001** | 0.815 | **<0.001** |
| **2** | -17.1 ± 21.5 | -6.5 ± 19.9 | **<0.001** | >0.999 | **0.001** |
| **3** | -16.3 ± 18.8 | -5.7 ± 18.2 | **<0.001** | >0.999 | **0.001** |
| **4** | -0.4 ± 19.4 | 4.9 ± 16.4 | 0.942 | **0.001** | 0.261 |

Values presented as mean ± standard deviation. Sidak post-hoc test p-values; p < 0.05; n = 18.

**Table S8. High Frequency (HF) reactivity to Stroop and cold pressor test (CPT) over time**

|  | **∆ HF ( Log ms^2^)** | | **Stroop vs.**  **0 min** | **CPT vs.**  **0 min** | **Stroop vs. CPT** |
| --- | --- | --- | --- | --- | --- |
| **Time (min)** | **Stroop** | **CPT** | **p-value** | **p-value** | **p-value** |
| **0** | -0.08 ± 0.77 | -0.08 ± 0.77 | - | - | >0.999 |
| **1** | -2.11 ± 1.52 | -0.26 ± 0.81 | **<0.001** | 0.993 | **<0.001** |
| **2** | -1.29± 1.20 | -0.09 ± 0.83 | **<0.001** | >0.999 | **<0.001** |
| **3** | -1.27 ± 1.06 | -0.00 ± 0.62 | **<0.001** | >0.999 | **<0.001** |
| **4** | 0.00 ± 0.88 | 0.09 ± 0.73 | >0.999 | 0.996 | 0.997 |

Values presented as mean ± standard deviation. Sidak post-hoc test p-values; p < 0.05; n = 18.
